# Um Caso de Doença Cardíaca Metastática Revelada após Infarto Agudo do Miocárdio e Pericardite

**DOI:** 10.36660/abc.20190534

**Published:** 2020-09-11

**Authors:** Sofia Torres, Mariana Vasconcelos, Carla Sousa, Antonio J. Madureira, Alzira Nunes, Maria Júlia Maciel

**Affiliations:** 1 Centro Hospitalar Universitário de São João Porto Portugal Centro Hospitalar Universitário de São João, Porto – Portugal

**Keywords:** Infarto do Miocárdio, Pericardite, Metástases Cardíacas, Neoplasias Pulmonares, Imagem Multimodal, Ressonância Magnética Cardíaca, Tomografia Computadorizada

## Introdução

As metástases no coração e pericárdio são muito mais comuns que os tumores cardíacos primários e geralmente estão associadas a mau prognóstico.^1 , 2^ Embora sejam mais frequentemente assintomáticas, as metástases cardíacas podem simular doenças cardíacas primárias, como síndromes coronárias agudas, insuficiência cardíaca congestiva e pericardite.^3 , 4^ O câncer de pulmão é a fonte mais frequente de doença cardíaca metastática, seja por extensão direta ou por uma combinação de disseminação linfática, hematogênica e transvenosa.^2 , 5^

## Relato de Caso

Apresenta-se um caso de um paciente do sexo masculino, 62 anos, fumante, com histórico médico de hipertensão e dislipidemia. Ele foi internado pela primeira vez devido a um infarto agudo do miocárdio com supradesnivelamento do segmento ST (IAMCSST) da parede lateral. Uma angiografia coronária emergente (realizada 2 horas após o início da dor torácica) revelou estenose de 80% da artéria coronariana descendente anterior esquerda (DAE) média, oclusão total da Dg1 (primeiro ramo diagonal da DAE) em seu óstio e estenose distal de 70% do ramo posterolateral da artéria coronariana circunflexa esquerda (PL). Foi realizada angioplastia com implantação de stent farmacológico na DAE e dilatação com balão da Dg1. Dias depois foi efetuada angioplastia com implantação de stent farmacológico na PL.

O ecocardiograma transtorácico mostrou função sistólica biventricular preservada com alterações da contractilidade das paredes anterior e lateral. O paciente permaneceu assintomático durante o restante tempo de internação e recebeu alta médica.

Dois meses após a alta, o paciente foi readmitido devido a dor torácica pleurítica, ECG anormal mostrando elevação difusa do segmento ST com concavidade superior e elevação da proteína C reativa (199mg/L) e da troponina I de alta sensibilidade (2953 ng/L). O ecocardiograma transtorácico exibiu função sistólica biventricular preservada, com as alterações da contractilidade previamente relatadas e derrame pericárdico de pequeno volume. Com base nessa apresentação, as hipóteses diagnósticas levantadas foram a de síndrome de Dressler *versus* outras causas de pericardite com lesão miocárdica associada.

**Figura 1:**
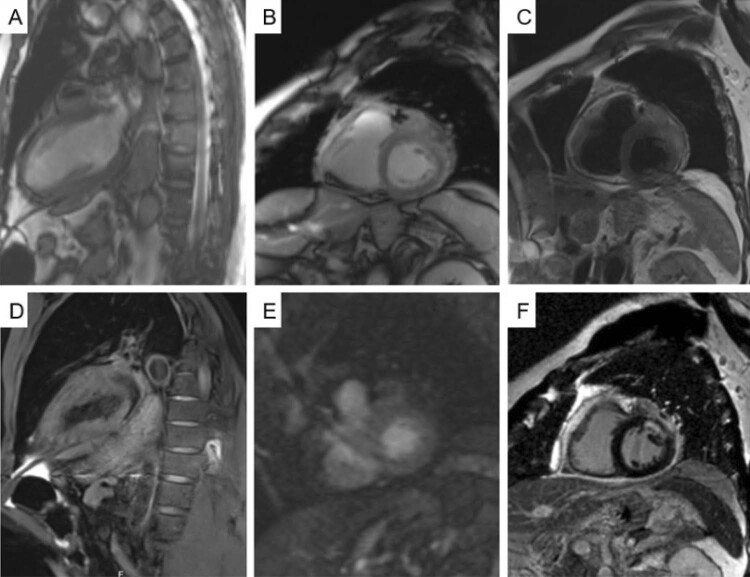
– RMC (A) (B) sequências cine b-SSFP revelando uma massa alongada (medindo 25 x 13 x 40 mm) adjacente aos segmentos basais anterior e antero-lateral, e em estreito contato com o stent na DAE. (C) Hipersinal nas sequências ponderadas em T2. (D) Isossinal nas sequências ponderadas em T1. (E) Perfusão de primeira passagem da massa. (F) Realce tardio com aspeto heterogéneo da massa e captação difusa do produto de contraste pelo pericárdio.

Foi realizada uma ressonância magnética cardíaca (RMC) para avaliação adicional, que revelou uma massa alongada intrapericárdica (medindo 25 x 13 x 40 mm) adjacente aos segmentos basais anterior e ântero-lateral e em estreito contato com o *stent* da DAE ( Figura 1 ). A massa apresentava sinal isointenso em imagens ponderadas em T1, hipersinal nas imagens ponderadas em T2, perfusão de primeira passagem e realce tardio heterogéneo após administração de gadolínio. A presença de realce tardio subendocárdico nos segmentos médio-basais anteriores e antero-laterais confirmou o infarto prévio no território da DAE. Também foi observada a presença de realce pericárdico, devido à presença de atividade inflamatória.

**Figura 2:**
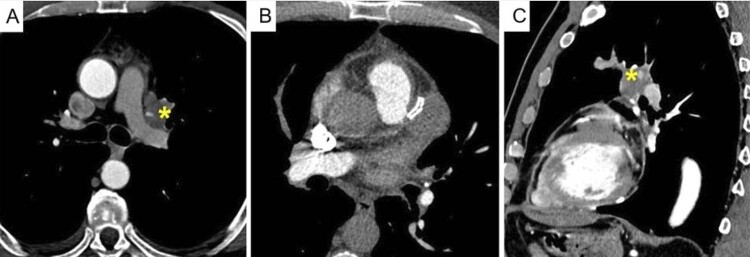
– TC tórax (A) (C) Lesão pulmonar suspeita (*) no hilo esquerdo, junto ao brônquio do lobo superior esquerdo e com invasão da veia pulmonar superior esquerda. (B) Massa metastática em estreito contato com o stent na DAE traduzindo envolvimento secundário do pericárdio.

Inicialmente, esses achados levantaram a preocupação de uma complicação do procedimento endovascular previamente realizado envolvendo a DAE, como dissecção coronária ou perfuração com hematoma organizado. Uma nova angiografia coronariana mostrou persistência do bom resultado em relação ao *stent* da DAE, sem sinais de complicações relacionadas com o procedimento. Suspeitou-se então de uma origem neoplásica da massa. Foi realizada uma tomografia computadorizada (TC) do tórax que revelou uma lesão suspeita no hilo esquerdo, junto ao brônquio do lobo superior esquerdo com invasão da veia pulmonar superior esquerda ( Figura 2 ). A biópsia da lesão pulmonar esquerda demonstrou um tumor carcinóide do pulmão.

A presença de linfadenopatias e nódulos pleurais apontou para uma natureza metastática da massa adjacente à DAE. A elevação da troponina de alta sensibilidade foi interpretada como relacionada com a infiltração miocárdica. Apesar da presença de doença aterosclerótica em outras artérias coronarianas, não foi possível excluir a hipótese de compressão externa da DAE pela massa metastática como contribuinte para o IAMCSST prévio da parede lateral.

O diagnóstico final foi de uma neoplasia pulmonar primária com envolvimento cardíaco secundário.

Investigações posteriores revelaram doença metastática generalizada com envolvimento ósseo, da glândula parótida, pancreático e cerebral e o paciente iniciou quimioterapia direcionada e radioterapia. Aos dois anos de acompanhamento, o paciente encontrava-se livre de sintomas e eventos cardíacos e permanecia sob tratamento quimioterápico paliativo.

## Conclusão

Os sintomas relacionados com a doença cardíaca metastática, que podem ser inespecíficos e mimetizar outros distúrbios cardíacos, como doença arterial coronariana ou pericardite, raramente representam a primeira manifestação de uma malignidade previamente desconhecida. Enquanto o ecocardiograma é o método de imagem mais utilizado para examinar o coração e o pericárdio, a imagem multimodal com RMC e TC oferece vantagens no diagnóstico da doença cardíaca metastática,^6 , 7^ como foi demonstrado neste caso.
