# Supplementary material for: Um Caso de Doença Cardíaca Metastática Revelada após Infarto Agudo do Miocárdio e Pericardite
Source: Arq Bras Cardiol. 2020 Sep 11;115(1 Suppl 1):22–4. [Article in Portuguese] doi: 10.36660/abc.20190534 (PMC8386951; doi:10.36660/abc.20190534)
Supplement: Translation

# Translation: A Case of Acute Myocardial Infarction and Pericarditis Unmasking Metastatic Involvement of the Heart

**Authors:** Sofia Torres, Mariana Vasconcelos, Carla Sousa, Antonio J. Madureira, Alzira Nunes, Maria Júlia Maciel

**Affiliations:** 1 Portugal Centro Hospitalar Universitário de São João, Porto – Portugal

**Keywords:** Myocardial Infarction, Pericarditis, Cardiac metastases, Lung Neoplasms, Multimodality Imaging, Cardiac Magnetic Ressonance, Computed Tomography

## Introduction

Metastases to the heart and pericardium are much more common than primary cardiac tumors and are generally associated with a poor prognosis.^1 , 2^ While they are most commonly asymptomatic, cardiac metastases can mimic primary cardiac diseases such as acute coronary syndromes, congestive heart failure and pericarditis.^3 , 4^ Lung cancer is the most frequent source of metastatic cardiac disease, either from direct extension or by a combination of lymphatic, hematogenous, and transvenous dissemination.^2 , 5^

## Case Report

We present a case of a 62-year-old male patient who had a medical history of hypertension and dyslipidemia and was a current smoker. He was first admitted to the hospital due to a lateral wall ST-segment elevation myocardial infarction (STEMI). Emergent coronary angiography (performed 2 hours after the onset of chest pain) revealed an 80% stenosis of the mid left anterior descending coronary artery (LAD), a total occlusion of the Dg1 (first diagonal branch of the LAD) on its ostium and a distal 70% stenosis of the left posterolateral branch of the left circumflex coronary artery (PL). Angioplasty with drug eluting stent (DES) implantation in the LAD and balloon dilatation of the Dg1 was performed. Percutaneous coronary intervention with DES implantation in the PL was conducted a few days later.

The transthoracic echocardiogram (TTE) showed preserved biventricular systolic function with anterior and lateral wall motion abnormalities. The patient remained asymptomatic afterwards and was discharged home.

Two months after discharge, the patient was readmitted due to pleuritic chest pain, abnormal ECG showing diffuse upward concave ST-segment elevation and elevated C-reactive protein (199 mg/L) and high-sensitive troponin I (2953 ng/L). The TTE exhibited preserved biventricular systolic function with the previously reported wall motion abnormalities and mild pericardial effusion. Based on this presentation, the diagnostic hypotheses raised were Dressler syndrome *versus* other causes of pericarditis with associated myocardial injury.

A cardiac magnetic resonance imaging (cMRI) was performed for further evaluation, which revealed an intrapericardial elongated mass (measuring 25 x 13 x 40 mm) adjacent to the basal anterior and anterolateral segments and in close contact with the LAD stent ( Figure 1 ). This mass had isointense signal intensity on T1-weighted images, high signal intensity on T2-weighted images, first-pass perfusion, and heterogeneous late gadolinium enhancement (LGE). Subendocardial LGE in the mid-basal anterior and anterolateral segments confirmed the previous infarction in the LAD territory. Contrast-enhanced pericardium was also noted, due to inflammatory activity.

**Figure 1:** – cMRI (A) (B) b-SSFP cine images revealing an elongated mass (measuring 25 x 13 x 40 mm) adjacent to the basal anterior and antero-lateral segments and in close contact with the LAD artery stent. (C) High intensity signal on T2-weighted images. (D) Isointense signal intensity on T1-weighted images. (E) First pass perfusion of the mass. (F) LGE with heterogeneous appearance of the mass and diffuse contrast-enhanced pericardium.

At first, these findings raised concerns about a complication of the previous endovascular procedure involving the LAD artery, such as coronary dissection or perforation with an organizing hematoma. A new coronary angiography showed persistence of the good result regarding the LAD stent, with no signs of procedure complications. A neoplastic origin of the mass was then suspected. A thoracic computed tomography (CT) was performed and unveiled a suspicious lesion in the left hilum, just next to the left superior lobe bronchus with invasion of the left superior pulmonary vein ( Figure 2 ). A biopsy of the left pulmonary lesion revealed a carcinoid tumor of the lung.

**Figure 2:** – Thoracic CT (A) (C) Suspicious pulmonary lesion (*) in the left hilum, just next to the left superior lobe bronchus with invasion of the left superior pulmonary vein. (B) Metastatic mass in close contact with the LAD stent traducing secondary involvement of the pericardium.

The presence of lymphadenopathy and pleural nodules pointed toward a metastatic nature of the mass adjacent to the LAD. High sensitive troponin elevation was interpreted as related to myocardial infiltration. Despite the presence of atherosclerotic disease in other coronary arteries, the hypothesis of external compression of the LAD by the metastatic mass as a contributor to the previous lateral wall STEMI could not be excluded.

The final diagnosis was a primary lung malignancy with secondary involvement of the heart.

Further investigation later unveiled widespread metastatic disease with bone, parotid gland, pancreatic and brain involvement and the patient started on targeted chemo- and radiotherapy. At two years of follow-up, the patient is free from cardiac symptoms and events and remains on palliative chemotherapy.

## Conclusion

Symptoms related to metastatic heart disease, which can be nonspecific and mimic other cardiac disorders such as coronary artery disease or pericarditis, can rarely be the first manifestation of a previously unknown malignancy. Whereas echocardiography is the most frequently used imaging method to examine the heart and pericardium, multimodality imaging with cMRI and CT offers advantages in the diagnosis of metastatic heart disease,^6 , 7^ as was demonstrated in this case.
